# Protocol for the THREAD (THREshold for AntiDepressants) study: a randomised controlled trial to determine the clinical and cost-effectiveness of antidepressants plus supportive care, versus supportive care alone, for mild to moderate depression in UK general practice

**DOI:** 10.1186/1471-2296-8-2

**Published:** 2007-01-04

**Authors:** Judy Chatwin, Tony Kendrick, the THREAD Study Group

**Affiliations:** 1 University of Southampton, School of Medicine, Aldermoor Health Centre, Aldermoor Close, Southampton SO16 5ST, UK; 2 Institute of Psychiatry, King's College, University of London, De Crespigny Park, Denmark Hill, London, SE5 8AF, UK; 3 The University of Liverpool, The Whelan Building, 2^nd ^Floor, Quadrangle, Brownlow Hill, Liverpool L69 3GB, UK

## Abstract

**Background:**

Depression guidelines in the UK recommended a policy of watchful waiting for mild depression due to a lack of evidence for the effectiveness of antidepressant treatment for mild cases. However there has been relatively little research carried out in primary care to help establish the severity threshold at which antidepressant treatment is effective and cost-effective.

**Methods/Design:**

The THREAD (THREshold for AntiDepressants) study is a multi-centre randomised controlled trial designed to determine the clinical and cost effectiveness of a selective serotonin reuptake inhibitor (SSRI) plus general practitioner (GP) supportive care, versus supportive care alone, for mild to moderate depression in primary care. The aim is to recruit 300 patients from three centres (Southampton, London and Liverpool). Depressive symptoms will be assessed at baseline, 12 weeks and 26 weeks, using the 17-item Hamilton Depression Rating Scale (HDRS). Two severity sub-groups of patients will be recruited, with HDRS scores of 12–15, and 16–19. Possible predictors of response will be explored including life events and difficulties and alcohol consumption. Analysis of covariance, controlling for baseline value, severity group and centre will be used to estimate the overall treatment effectiveness (difference in HDRS score) at final follow up. The primary analysis will be by 'intention to treat' using double sided tests. The interaction between severity sub-group and treatment will be tested, and if appropriate, effects within separate severity sub-groups estimated. The economic analysis will compare the two treatment groups in terms of mean costs and cost-effectiveness.

**Discussion:**

The results of this study will give GPs important information to help them determine the severity of depression at which antidepressant treatment is likely to be cost-effective.

## Background

### Increasing prescribing of antidepressants – is it appropriate?

Prescribing of antidepressant drugs has increased by 36% over the last 5 years to around 30 million items (7.3 million in the quarter to June 2005), and the cost has increased by 20% to around £380 million (£91 million for the same quarter) [1].

However, much of this increased prescribing may be inappropriate. As a result of the perceived pressure to treat more depression, antidepressants are being prescribed more frequently for depressive symptoms below the threshold for major depression [2]. Clinical guidelines recommend antidepressant medication as first-line treatment for patients meeting diagnostic criteria for major depressive disorder [3,4]. Antidepressants are not recommended for the initial treatment of mild depression because the risk-benefit ratio is considered to be poor. However, these guidelines have been produced based largely on consensus or expert opinion. There has been relatively little research in primary care on which to base recommendations on the threshold at which antidepressants should be offered. Studies in primary care have shown that antidepressants are more effective than placebo or treatment as usual for probable major depression but results have been mixed for minor (mild) depression.

### Previous research in primary care

A general practice based placebo-controlled trial of amitriptyline found that patients with probable major depressive disorder benefited from drug treatment, but those with minor depression did no better on them than on placebo [5]. However these findings represent a post-hoc analysis of responses in the two sub-groups of patients who did or did not fulfil criteria for a diagnosis of probable major depression. The study was not set up specifically to assess the relationship between severity and response to treatment.

A placebo-controlled trial of the SSRI paroxetine, versus problem-solving, versus non-specific clinical management or 'watchful waiting' for minor depression and dysthymia in a primary care population was undertaken in the USA [6]. The results were mixed: among patients aged 18 to 59 years with dysthymia, paroxetine improved remission compared with placebo plus non-specific clinical management, while for minor depression they were equally effective [7]. Among patients aged 60 and over paroxetine was beneficial in dysthymia and among more severely impaired patients with minor depression [8]. The authors suggested that 'watchful waiting' i.e. supportive care but without the prescription of antidepressants, was an appropriate treatment option for minor depression, at least in adults and in elderly patients with mild impairment.

Judd et al [9] carried out a randomised placebo controlled trial of fluoxetine among 162 patients with minor depressive disorder and found that fluoxetine was better in terms of clinical effectiveness in terms of the Hamilton Depression Rating Scale (HDRS). The mean difference was only one point on the HDRS. Minor depression in these subjects was primarily characterised by mood and cognitive symptoms, not the classical neurovegetative signs and symptoms. One third had a past history of major depressive disorder, however HDRS scores at baseline ranged from 6–21 inclusive, on the 17 item scale [10].

Finally, most recently, Perahia et al [11] found that duloxetine was more effective than placebo in 159 patients with milder major depressive disorder (HDRS scores on the HAMD17 between 15 and 18 inclusive) over 9 weeks. The mean different was relatively small, 2.9 points on the HDRS. However, this was a post-hoc sub-group analysis of pooled data from two trials.

There is evidence that one form of mild depression, dysthymia, responds to antidepressants. Dysthymia is a term used to describe chronic low-grade depression and in ICD-10 requires that four or more depressive symptoms are present for at least two years [12]. A recent systematic review and meta-analysis suggested that antidepressant drug treatment is effective in the management of dysthymia, but the research studies analysed were conducted in secondary care settings [13].

### Predictors of response

The placebo-controlled trial of amitriptyline in general practice referred to above found no difference between those categorised as having endogenous and non-endogenous depression. The authors recommended drug treatment for major depression, regardless of demographic characteristics, a past history of depression, or the presence or absence of endogenous features [14]. These findings have led to recommendations to prescribe drug treatment for depression if symptoms are severe enough, and functioning is impaired, even if there seems to be an understandable cause for depression such as adverse events or continuing difficulties in the patient's life [3,15-17]. However, the importance of social factors in depression is undeniable, and there is substantial evidence to suggest that both onset and recovery are related to life events and difficulties.

Depression is strongly associated with lower socioeconomic status [18,19], poverty [20], unemployment [19,21], separation or divorce [18,22], and poor housing [23]. Predisposing factors among women include demanding child care [24], lone motherhood and poor social support [25]. A lower severity of premorbid difficulties has been shown to be associated with a reduced time to remission, at least among patients with high self-esteem and better coping strategies [26]. Recovery from depression is related to positive social support and life events which can be perceived as 'fresh starts', which may or may not be related to the original adverse events and difficulties associated with onset [27]. A reduction in marked social difficulties has been found to predict recovery from depression among patients in primary care [28] whereas recognition and drug treatment by the general practitioner has not [29,30].

The scant research into psychosocial predictors of response carried out so far in secondary care suggests that greater emotional support, and a relative lack of experience of adversity, particularly in domains of the patient's life which are regarded as most important, are more strongly related to recovery than drug treatment [31]. As findings in secondary care may not generalise to primary care however, more research is needed to determine whether such social and cognitive factors predict response to drug treatment in a primary care setting.

In general somatic presentations of depressive disorder are associated with reduced severity of depressive symptoms but similar impairments in function and a similar prognosis [32]. However, it is important to go on to explore whether the number of physical symptoms affects treatment responses when comparing drug and non-drug treatments.

### The need for a new study

The US studies described above compared SSRIs to placebo and were therefore efficacy trials which cannot establish cost-effectiveness in practice. Also, findings from the USA may not generalise to primary care in the UK, as the type of supportive care usually provided in the UK, which may include referral to a practice counsellor, differs from the 'watchful waiting' provided in the US study. If SSRI treatment is effective in mild depression, its cost-effectiveness needs to be established within the UK health care system. Predictors of response to antidepressant treatment also need to be identified, to help practitioners decide which patients should be offered them.

The THREAD study is designed to inform guideline recommendations by exploring in general practice whether the added prescription of a SSRI antidepressant is more cost-effective than support from the GP alone in patients with mild to moderate depression.

Our research questions are:

1. Is treatment with a SSRI plus supportive care more effective and cost-effective than supportive care alone?

2. If it is more effective, does this apply across the whole range of severity of symptoms of mild to moderate depression?

3. What patient factors might predict a beneficial response?

The hypothesis is that SSRI treatment will be more effective and cost-effective than supportive care alone among patients scoring 16–19 on the Hamilton Rating Scale (HDRS) but not among those scoring 12–15.

We will also explore the impact of a number of predictors on the outcome in the two treatment arms, namely demographic and social variables, alcohol consumption, physical symptoms, recent life events and difficulties, the patient's self-rating of the cause of their illness, and the duration of their depressive symptoms.

## Methods/Design

### Trial design

The study is a randomised controlled trial that compares treatment with a SSRI plus supportive care, versus supportive care alone, over 26 weeks follow-up. The aim is to establish the clinical and cost effectiveness of SSRI antidepressants added to GP support over GP support alone, in clinical practice conditions. It is not an efficacy study and there is no placebo control group (placebos are not given in clinical practice and cannot be costed into the economic analysis).

### Ethical approval

Ethical approval was gained from the West Midlands Multi-Centre Research Committee (MREC) (reference number: 02/7/091).

### Setting

Patients are being recruited from general practices around three centres: Southampton, London (co-ordinated by the Institute of Psychiatry and Kings College London), and Liverpool.

### Patients

#### Inclusion criteria

Patients are eligible for inclusion if they are aged 18 and above, and are found to be depressed by their GPs and potentially in need of treatment. Patients must have at least one somatic symptom on the Bradford Somatic Inventory [33]. Only patients for whom the likely benefit of treatment is uncertain in the mind of the GP are referred to the study because it is essential that the GP is in equipoise about the likely outcome. To avoid including patients with more transient depression, they need to have had symptoms for at least eight weeks. To include only patients with new episodes of depression, they must not have received treatment for depression within the previous 12 months.

#### Exclusion criteria

Patients are excluded from the study if:

• they are found to have an HDRS score of less than 12, or greater than 19

• they suffer from suicidal intent

• they report significant substance misuse, determined using screening questions

• if they score 13 or more on the Alcohol Use Disorders Identification Test (AUDIT) questionnaire

• they do not have the spoken or written language skills necessary to take part

**Figure 1:**
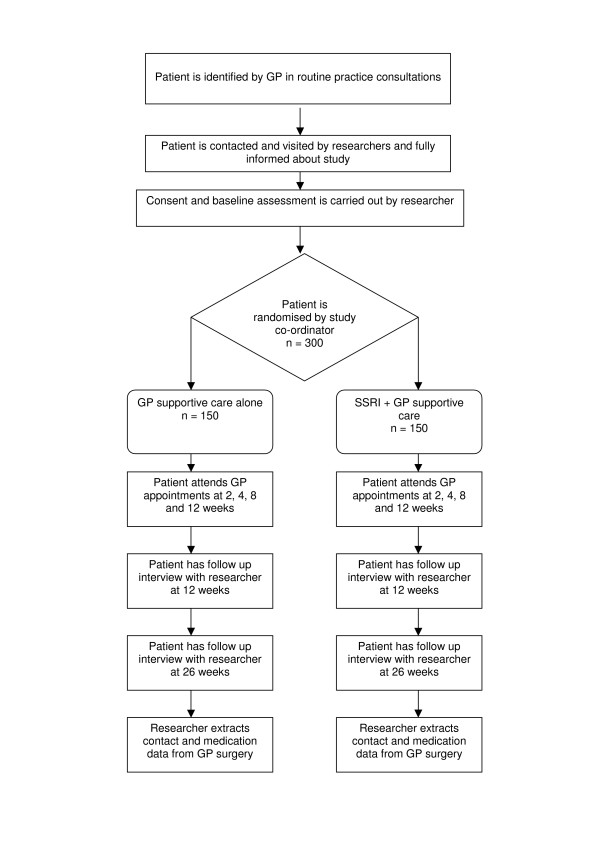
**Trial design**. Flow chart to show the study procedure.

Figure 1 shows the procedure of what is involved for the patients recruited into the study.

### Interventions

#### Supportive care alone

The GP is asked to see the patient for support in follow-up appointments at 2, 4, 8 and 12 weeks after they have been randomised into the study. They are asked to refrain from prescribing antidepressants during this 12 week period. However, if the patient deteriorates and the GP feels that they are in need of antidepressants then they can be started on drug treatment. If this does occur then the patient can continue in the study but remains in the support alone arm on an intention to treat basis.

#### SSRI antidepressant plus supportive care

The patient is prescribed an appropriate SSRI and then seen by the GP at the times listed above. The GP is also at liberty to switch SSRIs should the initial choice prove unsuitable. The GP is advised to continue treatment for four months after recovery but it is stressed to the GP that this is a pragmatic study and that they should use their clinical judgement in relation to the duration of the patient's treatment.

The GPs are free to refer patients in either arm for counselling or psychological therapy if this is appropriate in their judgement, but waiting times for counselling and psychological treatment are such that patients will not usually receive this before the 12 week follow-up, and so this will not affect the comparison for the primary outcome.

### Randomisation

Block randomisation with random block sizes, stratified by severity sub-group and centre, is carried out by an independent centre (The Institute of Psychiatry Clinical Trials Unit) over the telephone.

### Blindness

The researchers carrying out the interviews are kept blind to the treatment arm, if possible. Patients are asked not to reveal whether or not they have been prescribed antidepressants prior to the follow-up interviews. All instances of unblinding are recorded.

### Patient assessments

The initial assessment takes place at baseline with follow up interviews being carried out at 12 and 26 weeks after randomisation. Table 1 summarizes the measures that are used at each point.

**Table 1:** Summary of measures

**Measure**	**Baseline**	**12 week follow-up**	**26 week follow-up**
17-item Hamilton Depression Rating Scale Interview (HDRS)			
Sociodemographic questionnaire			
AUDIT measure of alcohol consumption			
Beck Depression Inventory (BDI-II)			
Date of onset questionnaire			
Short Form 36 (SF36) – health-related quality of life measure			
Bradford Somatic Inventory (BSI)			
Short Life Events and Difficulties Schedule (S-LEDS)			
Symptom attribution questionnaire			
Patient expectations questionnaire			
Client Service Receipt Inventory (CSRI)			
Patient Satisfaction Scale			
Measure of Care Received questionnaire			

A summary of which measures are collected from the patients at each time point during their participation in the study.

### Outcome measures

The primary outcome measure is the 17-item HDRS score at 12 weeks. The BDI-II, the SF-36, the Patient Satisfaction Scale and the HDRS at 26 weeks are all secondary outcomes. The sociodemographic, duration of depression, alcohol consumption, somatic symptom inventory, LEDS and symptom attribution measures are all potential predictors of response.

### Depressive symptoms

The Hamilton Depression Rating Scale (HDRS) is used to measure depressive symptoms as the primary outcome. A large number of studies have shown it to be a valid and reliable measure of depression [34]. Also it is sensitive to changes due to drug effects in a general practice setting [35]. The structured 17-item interview version of the HDRS is being used in this study and all the researchers involved have received extensive and on-going training in this measure [36]. Inter-rater reliability of ratings on the HDRS is being assessed at intervals throughout the recruitment and follow-up period.

The Beck Depression Inventory second edition (BDI-II) is a second measure of depressive symptoms [37]. This is a 21-item self-report rating inventory measuring characteristic attitudes and symptoms of depression. As this is self-complete it should be essentially free of any observer bias which will enable a check to be carried out to ensure that there is no systematic bias in the HDRS ratings arising from possible un-blinding of the researchers to treatment arm.

### Quality of life

The SF-36 is a questionnaire consisting of 36 items concerning respondents' health-related quality of life [38]. The responses to the items can be condensed into scores on eight domains of health-related quality of life: physical functioning, role-physical, role-emotional, social functioning, pain vitality, mental health and general health. The SF-36 will be used to calculate quality-adjusted life years (QALYs) to be used in the cost-effectiveness or cost utility analysis.

### Short life events and difficulties scale

This instrument is usually used to collect information about stressful experiences over a one-year period before onset/relapse of disorder. It differs from many other stress measures by distinguishing acute from ongoing stressors (events from difficulties), and by contrasting short and long term, and contextual and subjective ratings of these experiences. Specific qualitative aspects of stress such as losses, dangers, humiliations, entrapments, challenges and goal frustrations are also deliberately contrasted [39]. The shortened version of the LEDS, used in this study, is essentially the same interview process but the ratings concentrate on those events that are considered severe, with marked or moderate threat to the individual, and not on those which are deemed to carry only some or little threat, unless they are a fresh start experience of the type found to predict depressive remission.

### Alcohol consumption

The Alcohol Use Disorders Identification Test (AUDIT) is a 10-item questionnaire designed by the World Health Organisation to screen for hazardous alcohol intake in primary health care settings. It has high sensitivity and specificity and can be self-completed or administered in 2–4 minutes [40].

### Somatic symptoms

The Bradford Somatic Inventory is a 46 item questionnaire about symptoms experienced in the last month, which was especially designed to detect physical symptoms commonly found in depressed patients [33].

### Patient satisfaction

This is a 7-point Likert rating scale to determine the patient's satisfaction with the care they have been given. This includes the doctor's explanation of the illness and its seriousness, whether the doctor told the patient what they wanted to know, the doctor's interest in the person, warmth, friendliness, treatment of the patient as an equal, understanding, relief of problems relief of worries, and whether the patient understood how to follow the doctor's advice. This questionnaire was developed by the Primary Medical Care Group at Southampton.

### Care received

This is being measured at 12 and 26 weeks using a self-report questionnaire that was specifically designed for this study to provide a measure of the content of the consultations in order to determine whether supportive care was comparable in both arms. It includes 4 questions devised by Morisky et al [41] which are used to measure patient adherence to the medication for those in the antidepressant treatment arm.

### Economic evaluation

#### Client Service Receipt Inventory

Service use is measured comprehensively using a modified version of the Client Service Receipt Inventory (CSRI) [42] at baseline and 26 weeks. This will allow the economic impact of the different treatment options to be ascertained. Services measured include all contacts with GPs, other primary care professionals, psychiatrists, psychologists, community mental health nurses, counsellors, social care professionals and complementary therapy.

#### Analysis of costs

Costs are calculated using data collected through the CSRI at the 12 and 26 week follow ups. Service use and medication data which are collected from the surgery records will be pooled with the CSRI data to maximise completeness. These will be multiplied by standard unit cost data to generate service costs for each patient.

### Sample size

The sample will be divided into two severity sub-groups (HDRS scores 12–15 and 16–19). Hollyman *et al *found the standard deviation (SD) of the HDRS to be around 3.5, and reported roughly similar numbers of patients in these two severity ranges [43]. We assume this SD, equal numbers in the two sub-groups, a pre-post correlation of 0.5, and significance level of 0.05.

Using analysis of covariance controlling for baseline values, 49 patients at follow-up in each treatment/severity combination will allow the following effects to be detected (standard effect sizes in brackets): an overall average difference in HDRS score of 1.4 (0.4) with 90% power; an interaction (difference between effects in the two severity sub-groups) of 2.5 (0.7) with 80% power; and a difference between treatment arms within the more severe group of 2.0 (0.6) with 90% power. The latter two calculations are conservative (tending to underestimate the power) because the SDs may be lower within the severity subgroups. A difference of 1.4 on the HDRS is a relatively small difference (0.4 standard deviations) and any difference smaller than this we regard as clinically insignificant. In the trial of amitriptyline by Hollyman *et al *the HDRS scores fell by a mean of around 10 points in the mildly depressed group and around 13 in the more severely affected group [43]. Therefore the sample size should be sufficient to detect clinically significant differences.

One hundred and ninety six patients will therefore need to be followed up to detect these differences (98 in each of the two arms). To allow for up to 25% loss to follow-up at 12 weeks, 261, 87 at each of the three sites, would be needed. The aim is to recruit 300, 100 at each site.

### Analysis

Analysis of covariance, controlling for baseline value, severity sub-group and centre will be used to estimate the overall treatment effectiveness (difference in HDRS score) at follow up. The primary analysis will be by 'intention to treat' using double sided tests. The interaction between severity sub-group and treatment will be tested, and if appropriate, effects within separate severity sub-groups estimated. Further exploratory analyses will also assess the impact of other explanatory factors and will also model the time course of effects using the 12-week measurement in a panel analysis. Sensitivity analyses will include estimating 'on treatment' effects and CACE (complier average causal effects), and by imputation of any missing values.

The main aim of the economic analysis is to compare the two treatment groups in terms of mean costs and cost-effectiveness. A secondary aim is to examine differences between the sub-groups defined by severity. Cost data are frequently skewed and this can cause a violation of the assumptions of standard significance tests. In the event of this, bootstrapped estimates (multiple re-sampling within treatment arms) will be determined, whereby mean costs can still be compared whilst imposing no prior assumptions regarding the data distribution. To explore cost-effectiveness, a net benefit variable will be generated which synthesises data on costs and outcomes, also analysed by bootstrapping.

## Discussion

It is intended that this study will provide the evidence needed to enable GPs to decide which of their patients suffering from mild and moderate depression are most likely to benefit from taking an SSRI antidepressant with regard to severity as well as other predictors.

## Competing interests

Tony Kendrick has received fees for presenting at educational meetings, and/or research grant funding, from Lilly, Lundbeck, Servier, and Wyeth pharmaceuticals. Judy Chatwin declares that she has no competing interests.

Members of the THREAD Study Group have declared the following competing interests.

Robert Peveler has received fees for speaking and/or consultancy from Lilly, Glaxo Smith Kline, Pfizer, Lundbeck, Wyeth, Astra Zeneca, Bristol Myers Squibb and Organon. Andre Tylee has received fees for presenting at educational meetings and on research funding from: Lilly; Lundbeck; Servier: Wyeth and Glaxo Smith Kline. Christopher Dowrick declares that he has no competing interests. Morven Leese declares that she has no competing interests.

Tom Craig declares that he has no competing interests. Tirril Harris declares that she has no competing interests. Paul McCrone has received fees for speaking and/or consultancy from Lilly, Lundbeck, Organon, Servier and Janssen-Cilag. Michael Moore declares that he has no competing interests. Richard Byng has received fees from Lilly. Richard Morriss declares that he has received fees for presenting from Lilly and Astra Zeneca. Mark Gabbay declares that he has no competing interests. Anthony Mann has not declared any competing interests. George Brown has not declared any competing interests.

## Authors' contributions

TK was instrumental in the design of the study, the writing of the protocol, chairing the study management group, contributing to the data analysis and will write up the results. He also read and approved the final manuscript. JC has co-ordinated the study, overseen the accurate collection of data and prepared the final manuscript for submission.

## Pre-publication history

The pre-publication history for this paper can be accessed here:


